# Adherence to Antiretroviral Therapy and Clinical Outcomes Among Young Adults Reporting High-Risk Sexual Behavior, Including Men Who Have Sex with Men, in Coastal Kenya

**DOI:** 10.1007/s10461-013-0445-9

**Published:** 2013-03-15

**Authors:** Susan M. Graham, Peter Mugo, Evanson Gichuru, Alexander Thiong’o, Michael Macharia, Haile S. Okuku, Elise van der Elst, Matthew A. Price, Nicholas Muraguri, Eduard J. Sanders

**Affiliations:** 1 Departments of Medicine and Global Health, University of Washington, Box 359909, 325 Ninth Avenue, Seattle, WA 98104-2499 USA; 2 Centre for Geographic Medicine Research – Coast, Kenya Medical Research Institute, Kilifi, Kenya; 3 International AIDS Vaccine Initiative, New York City, NY USA; 4 National AIDS and STI Control Programme, Nairobi, Kenya; 5 Nuffield Department of Clinical Medicine, University of Oxford, Oxford, England UK

**Keywords:** Men who have sex with men, Kenya, Antiretroviral therapy, Adherence, Retention, Los hombres que tienen sexo con hombres, Kenya, Tratamiento antirretroviral, Adhesión, Retención

## Abstract

African men who have sex with men (MSM) face significant stigma and barriers to care. We investigated antiretroviral therapy (ART) adherence among high-risk adults, including MSM, participating in a clinic-based cohort. Survival analysis was used to compare attrition across patient groups. Differences in adherence, weight gain, and CD4 counts after ART initiation were assessed. Among 250 HIV-1-seropositive adults, including 108 MSM, 15 heterosexual men, and 127 women, patient group was not associated with attrition. Among 58 participants who were followed on ART, 40 % of MSM had less than 95 % adherence, versus 28.6 % of heterosexual men and 11.5 % of women. Although MSM gained less weight after ART initiation than women (adjusted difference −3.5 kg/year), CD4 counts did not differ. More data are needed on barriers to adherence and clinical outcomes among African MSM, to ensure that MSM can access care and derive treatment and prevention benefits from ART.

## Resumen

En África los hombres que tienen sexo con hombres (HSH) sufren un nivel importante de estigma y encuentran obstáculos a la atención médica. Investigamos la adhesión al tratamiento antirretroviral (TAR) en un grupo de adultos de alto riesgo (el cual incluyó HSH) que participó en una cohorte de una clínica. Utilizamos el análisis de supervivencia para comparar el abandono de participantes en los distintos grupos. Evaluamos las diferencias en la adhesión al TAR, aumento de peso, y los recuentos de CD4 desde el inicio de TAR. En los 250 adultos con VIH-1, que incluyeron 108 HSH, 15 hombres heterosexuales y 127 mujeres, no observándose abandono. De los 58 participantes que tuvieron seguimiento mientras recibían TAR, el 40 % de los HSH tuvo <95 % de adhesión, versus el 28.6 % de los hombres heterosexuales y el 11.5 % de las mujeres. Aunque los HSH subieron menos peso que las mujeres después del inicio del TAR (diferencia ajustada −3.5 kg/año), el recuento de CD4 no fue diferente. Concluimos que es necesario profundizar la investigación sobre las asociaciones entre las barreras a la adhesión al TAR y los resultados clínicos en los HSH de África, para garantizar que ellos tengan acceso a la atención médica y así beneficiarse del tratamiento y de la prevención que provee el TAR.

## Introduction

Sub-Saharan Africa has a very high burden of HIV-1 infection, of which a substantial proportion (estimated at 29 % in Kenya) occurs among populations reporting high-risk sexual behavior such as transactional sex and anal intercourse [1]. Such populations suffer from stigma and discrimination, and have been neglected by many HIV prevention and care programs [1]. In particular, men who have sex with men (MSM) have only recently become a focus for HIV programs in this region [1–4].

Because MSM are highly stigmatized by prevailing attitudes and laws against adult same-sex behavior in most African countries [5], they may be reluctant to access care and lack trust in providers [6, 7]. Access to antiretroviral therapy (ART), retention in care, and adherence to treatment are critical determinants of health outcomes among HIV-1-infected individuals [8–12]. In addition, ART prevents transmission to the sexual partners of treated individuals [13, 14], and high uptake could significantly decrease new infections among MSM and on a population level [13, 15]. However, little is known about antiretroviral adherence among this group.

We and others have identified MSM as an important HIV risk group on the Kenyan coast [16–18]. Since 2004, we have conducted HIV prevention research and offered ART to eligible HIV-1-infected individuals from high-risk groups including MSM, female sex workers (FSW), and persons with multiple sexual partners. We describe here our experience in providing ART to this population. We hypothesized that Kenyan MSM would have delayed engagement in care, lower ART adherence, and worse clinical outcomes compared to high-risk men and women participating in the same HIV-1-seropositive cohort.

## Methods

### Study Population

The study population consisted of participants in a prospective cohort based in a research clinic north of Mombasa, Kenya. Adults aged 18–49 years were eligible if they resided in the clinic catchment area (i.e., the coastal region from Kilifi in the north to Mombasa in the south), were HIV-1-seropositive, and reported transactional sex work, multiple sexual partners, anal sex, or a recent sexually transmitted infection (STI). HIV-1-seronegative persons enrolled in a parallel clinic-based cohort that has been described previously [17]. Identification and recruitment of potential study participants was carried out by trained peer mobilizers, who approached individuals via personal networks and at venues where high-risk sex was common.

### Clinical Procedures

Eligibility screening and cohort enrollment were carried out by trained research staff with experience in HIV counseling and testing. These staff conducted all research visits for participants in the two cohorts (i.e., HIV-seropositive and HIV-seronegative) according to established protocols. At enrollment, participants completed face-to-face or audio computer-assisted interviews using standardized questionnaires to record recent sexual behavior and health status. A standardized physical exam was performed, and specimens were collected for STI screening. Participants with genital symptoms were provided syndromic treatment, and laboratory-diagnosed infections were treated following Kenyan Ministry of Health guidelines. Risk reduction counseling, condoms, and lubricants were provided. These procedures were repeated at quarterly follow-up visits.

At enrolment and each subsequent visit, participants were reviewed for ART eligibility according to Kenyan National Guidelines during this period (CD4 count ≤250 cells/μL or AIDS-defining illness). All eligible participants were offered ART at the time of eligibility. Data on ART status were recorded at each visit. Neither ART eligibility nor uptake was required for cohort participation, and cohort participants were free to initiate or continue ART at other local clinics if they preferred. All clinic attendees were cohort participants, and cohort withdrawal resulted in transfer of care to another clinic.

The standard ART regimen consisted of stavudine, zidovudine, or tenofovir with lamivudine and either nevirapine (for women) or efavirenz (for men), according to Kenyan National Guidelines. Monthly support groups were used to promote adherence, which was monitored at each refill by a visual analog scale (VAS) [19]. Refill dates and the quantity of drug dispensed were also recorded. Participants taking ART through the research clinic were tracked via telephone or in person after any missed visit or refill; cohort participants not taking ART were not routinely tracked.

### Ethical Approval

The study was approved by ethical review boards at the Kenya Medical Research Institute and University of Washington. All participants provided written informed consent.

### Laboratory Testing

HIV-1 status was confirmed using two rapid test kits in parallel (Determine, Abbott Laboratories, Abbott Park, Illinois, USA; Unigold, Trinity Biotech, Bray, Ireland). Discrepant rapid HIV-1 test results were resolved using an ELISA (Genetic System HIV-1/2 plus O EIA, Bio-Rad Laboratories, Redmond, Washington, USA). CD4 counts (FACS Count, Becton–Dickinson) were determined every 3 months if the last count was <350 cells/μL and every 6 months if ≥350 cells/μL. Plasma viral loads were not available.

### Definitions

Men who have sex with men were defined as men who reported sex with another man either at enrollment or subsequently during follow-up. Heterosexual men were men who never reported a male sexual partner during cohort participation. Nutritional status was categorized using the calculated body mass index (BMI) as adequate (BMI ≥ 18.5), mild or moderate malnutrition (BMI 16–18.49), or severe malnutrition (BMI < 16) [20]. CD4 counts were categorized as <200 cells/μL, 200–350 cells/μL, and >350 cells/μL following WHO guidelines for ART initiation (i.e., <200 prior to 2010, ≤350 currently) [21]. Loss to follow-up (from cohort and clinic) was defined as a final visit ≥12 months before March 1, 2011, with no formal notice of withdrawal. Cohort withdrawal was defined as having formally transferred care elsewhere.

Adherence measures were calculated using primary data from clinic visits (i.e., VAS result, refill dates, pills dispensed). VAS adherence was 100 % minus the percentage of pills reported missed over the last 30 days [19]. Adherence by pharmacy records was calculated as the percentage of days since the last refill on which a pill supply was available. Overall adherence was the lower of these two measures. A late or missed refill was defined as any refill provided after the due date, which was scheduled for the day on which the pill supply would be exhausted, assuming no missed doses. Loss to follow-up from the clinic’s ART program was defined as having missed ≥3 consecutive monthly refills before the study end date.

### Statistical Analysis

Pearson’s χ^2^ or Fisher’s exact (FE) tests were used to compare categorical predictors across patient groups at baseline and ART initiation. The Kruskal–Wallis (KW) equality-of-populations rank test was used to compare non-normally distributed data (e.g., follow-up time) across groups. Kaplan–Meier curves were used to characterize trends in loss to follow-up and in mortality over time stratified by group, and log-rank tests were used to test for differences in these curves. Cox regression analysis was used to evaluate the association between patient group and loss to follow-up, after adjustment for potential confounding factors. For this analysis, data were censored at the first of cohort withdrawal, death, or March 1, 2011. ART status was categorized as not taking ART, taking ART prescribed by the research clinic, or taking ART prescribed by an outside clinic. Potential confounding factors included characteristics recorded at enrolment (e.g., marital status) and time-dependent covariates updated at each visit (e.g., ART status). Covariates identified a priori (i.e., group, age category) and those with a *p* value <0.10 in bivariable analysis were selected for inclusion in multivariable models.

For an analysis of outcomes after ART initiation, only participants who started ART in the research clinic and had at least one on-treatment follow-up visit were included. Log-rank tests were used to test for differences in the hazard functions for loss to follow-up and mortality. Data from all visits after ART initiation were aggregated to calculate mean adherence and any late refills or adherence <95 %. These data were compared across patient groups, using KW equality-of-populations rank tests, Pearson’s χ^2^, or FE tests, as appropriate. Data from the first 12 months after ART initiation were analyzed to evaluate clinical outcomes including weight gain and CD4 count increase. Baseline values were the last measurements (within 159 days) prior to ART initiation, and on-treatment values were the last measurement between 180 and 365 days after ART initiation. This window was selected to maximize the number of participants with on-treatment assessment. Differences between baseline and on-treatment weight and CD4 count were calculated, then divided by the time in years after ART initiation, to account for differences in each participant’s assessment time-point. Linear regression was then performed to identify associations between patient group and weight gain or CD4 increase. Potential confounders identified a priori (i.e., age category, prior therapy, second-line regimen) and those with a *p* value < 0.10 in bivariable analysis were selected for inclusion in multivariable models.

A two-sided *p* value < 0.05 was significant. Stata/IC version 12.1 (StataCorp LP, College Station, Texas) was used for all analyses.

## Results

### Cohort at Baseline

Between July 20, 2005 and March 1, 2011, 250 HIV-1-seropositive participants enrolled, including 127 women and 123 men, of whom 108 were classified as MSM and 15 as heterosexual for this analysis. Three men reported only female partners at enrollment, but reported sex with male partners at least once during follow-up, and so were classified as MSM. Table 1 compares demographic and health characteristics of participants at enrollment. There were significant differences across groups in marital status, having dependents, income, and transactional sex work. There were no differences in CD4 count, WHO stage, nutritional status, illness, or prior TB. MSM and heterosexual men were less likely than women to have disclosed their HIV status, used co-trimoxazole prophylaxis, had ART counseling, or ever taken ART at baseline, although differences in co-trimoxazole use were of borderline significance.

**Table 1:** Seropositive cohort at enrollment, *n* = 250

Characteristic	Overall *N* (%)	MSM^a^ (*n* = 108) *N* (%)	Heterosexual men^a^ (*n* = 15) *N* (%)	Women (*n* = 127) *N* (%)	χ^2^ statistic or FE	*p* value
Sex of sexual partners reported at enrollment						
Opposite sex	140 (56.0)	3 (2.8)	15 (100)	122 (96.1)	FE	<0.001
Both men and women	45 (18.0)	41 (38.0)	0	4 (3.1)		
Same sex	65 (26.0)	64 (59.3)	0	1 (0.8)		
Age group						
18–24 years	52 (20.8)	26 (24.1)	1 (6.7)	25 (19.7)	FE	0.28
25–34 years	143 (57.2)	64 (59.3)	10 (66.7)	69 (54.3)		
35 and older	55 (22.0)	18 (16.7)	4 (26.7)	33 (26.0)		
Marital status						
Single	164 (65.6)	89 (82.4)	7 (46.7)	68 (53.5)	FE	<0.001
Married	28 (11.2)	11 (10.2)	8 (53.3)	9 (7.1)		
Widowed, separated, or divorced	58 (23.2)	8 (7.4)	0	50 (39.4)		
Has dependents	136 (54.4)	31 (28.7)	12 (80.0)	93 (73.2)	FE	<0.001
Educational achievement						
None or primary school only	147 (58.8)	63 (58.3)	10 (66.7)	74 (58.3)	FE	0.53
Secondary school	85 (34.0)	36 (33.3)	3 (20.0)	46 (36.2)		
Higher education	18 (7.2)	9 (8.3)	2 (13.3)	7 (5.5)		
Monthly earnings^b^						
≥10,000 KSh	38 (15.2)	11 (10.2)	3 (20.0)	24 (18.9)	FE	0.015
5,000–9,999 KSh	63 (25.2)	30 (27.8)	8 (53.3)	25 (19.7)		
2,000–5,000 KSh	93 (37.2)	38 (35.2)	4 (26.7)	51 (40.2)		
<2,000 KSh	56 (22.4)	29 (26.8)	0	27 (21.3)		
Transactional sex	187 (74.8)	76 (70.4)	3 (20.0)	108 (85.0)	FE	<0.001
Alcohol use	166 (66.4)	67 (62.0)	9 (60.0)	90 (70.9)	2.33	0.31
CD4 count						
<200	34 (13.6)	17 (15.7)	1 (6.7)	16 (12.6)	FE	0.38
200–350	63 (25.2)	24 (22.2)	7 (46.7)	32 (25.2)		
>350	153 (61.2)	67 (62.0)	7 (46.7)	79 (62.2)		
WHO stage^c^						
I asymptomatic/lymphadenopathy	100 (40.5)	50 (46.7)	6 (40.0)	44 (35.2)	FE	0.14
II mild local manifestations	91 (36.8)	41 (38.3)	4 (26.7)	46 (36.8)		
III systemic manifestations	54 (21.9)	15 (14.0)	5 (33.3)	34 (27.2)		
IV AIDS-defining illness	2 (0.8)	1 (0.9)	0	1 (0.8)		
Nutritional status						
Severe malnutrition	3 (1.2)	2 (1.8)	0	1 (0.8)	FE	0.71
Mild to moderate malnutrition	47 (18.8)	23 (21.3)	3 (20.0)	21 (16.5)		
Adequate nutrition	200 (80.0)	83 (76.8)	12 (80.0)	105 (82.7)		
Too sick to work, past 3 months						
No	209 (83.6)	92 (85.2)	12 (80.0)	105 (82.7)	FE	0.76
Yes	41 (16.4)	16 (14.8)	3 (20.0)	22 (17.3)		
Prior TB^c^						
No	194 (79.8)	86 (82.7)	12 (80.0)	96 (77.4)	FE	0.60
Yes	49 (20.2)	18 (17.3)	3 (20.0)	28 (22.6)		
Disclosure of HIV status^c,d^						
No	141 (56.8)	73 (67.6)	8 (57.1)	60 (47.6)	9.46	0.009
Yes	107 (43.2)	35 (32.4)	6 (42.9)	66 (52.4)		
Cotrimoxazole use^c^						
No	193 (80.1)	89 (84.8)	14 (93.3)	90 (74.4)	FE	0.07
Yes	48 (19.9)	16 (15.2)	1 (6.7)	31 (25.6)		
Prior ART counseling^c^						
No	204 (83.6)	96 (89.7)	14 (93.3)	94 (77.0)	FE	0.02
Yes	40 (16.4)	11 (10.3)	1 (6.7)	28 (23.0)		
Prior ART use^c^						
No	208 (88.5)	96 (93.2)	15 (100)	97 (82.9)	FE	0.02
Yes^e^	27 (11.5)	7 (6.8)	0	20 (17.1)		

*FE* Fisher’s exact test significance value

^a^Participants were classified as MSM or heterosexual men based on behavior reported at enrolment or during follow-up

^b^1,000 KSh is approximately $11.90

^c^Data were missing for WHO stage (1 MSM, 2 women), prior TB (4 MSM, 3 women), disclosure (1 heterosexual man, 1 woman), cotrimoxazole use (3 MSM, 6 women), prior ART counseling (1 MSM, 5 women), and prior ART use (5 MSM, 10 women)

^d^Disclosure was defined as informing anyone other than the counselor or medical staff (e.g., family, partner, friend, other) about one’s HIV infection

^e^Includes 5 women who had taken ART for PMTCT

### Loss to Follow-Up

**Fig. 1:**
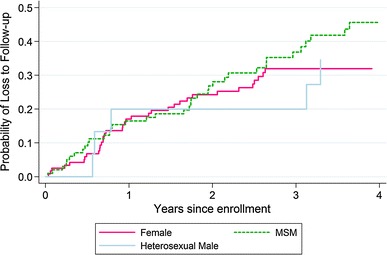
Kaplan–Meier curves for the probability of loss to follow-up after cohort enrollment by patient group (female, MSM, or heterosexual male)

After the initial visit, eight MSM and eight women did not return, and one MSM withdrew; these 17 participants therefore had no follow-up visits. Among 233 participants with at least one follow-up visit, 57 MSM (57.6 %), 10 heterosexual men (66.7 %), and 81 women (68.1 %) remained in follow-up on March 1, 2011. Two MSM (2.0 %) and three women (2.5 %) had died; 37 MSM (37.4 %), 5 heterosexual men (33.3 %), and 35 women (29.4 %) were lost to follow-up; and 3 MSM (3.0 %) had withdrawn from the clinic. In total, 77 participants were lost to follow-up from the cohort over 646.3 person-years, for a rate of 11.9 patients lost (95 % confidence interval [CI], 9.5–14.9) per 100 person-years of observation. Figure 1 presents the Kaplan–Meier failure curves for loss to follow-up in each group. Although MSM had the highest rate of loss to follow-up, this difference was not significant (14.5 per 100 person-years for MSM, 9.2 per 100 person-years for heterosexual men, and 10.4 per 100 person-years for women; χ^2^ = 1.60, *p* = 0.45 by log-rank test). Table 2 presents a multivariable analysis of factors associated with loss to follow-up. Younger participants were more likely (adjusted hazard ratio [aHR] 1.90, 95 % CI 1.00–3.62 if aged 18–24 years relative to ≥35 years), and participants receiving ART through the research clinic were less likely (aHR 0.42, 95 % CI 0.19–0.94) to be lost to follow-up.

**Table 2:** Associations with loss to follow-up after enrollment, *n* = 233

Characteristic	Baseline *N* (%)	HR (95 % CI)	*p* value	aHR (95 % CI)^a^	*p* value
Risk group					
Female	119 (51.1)	Reference		Reference	
MSM	99 (42.5)	1.33 (0.84–2.11)	0.23	1.33 (0.83–2.14)	0.24
Heterosexual males	15 (6.4)	0.97 (0.38–2.47)	0.94	1.12 (0.44–2.87)	0.82
Age group^b^					
18–24 years	49 (21.0)	2.24 (1.21–4.16)	0.01	1.90 (1.00–3.62)	0.05
25–34 years	133 (57.1)	0.83 (0.48–1.41)	0.49	0.72 (0.42–1.25)	0.24
35 and older	51 (21.9)	Reference		Reference	
Marital status					
Single	154 (66.1)	Reference			
Married	26 (11.2)	1.11 (0.58–2.14)	0.74		
Widowed, separated, or divorced	53 (22.8)	0.92 (0.53–1.62)	0.78		
Has dependents	127 (54.5)	0.79 (0.50–1.23)	0.30		
Educational achievement					
None or primary school only	141 (60.5)	Reference			
Secondary school	76 (32.6)	0.88 (0.54–1.43)	0.60		
Higher education	16 (6.9)	0.75 (0.27–2.08)	0.58		
Monthly earnings					
≥10,000 KSh	35 (15.0)	Reference			
5,000–9,999 KSh	60 (25.8)	1.61 (0.77–3.38)	0.21		
2,000–5,000 KSh	84 (36.0)	0.99 (0.47–2.09)	0.99		
<2,000 KSh	54 (23.2)	1.46 (0.69–3.10)	0.32		
Transactional sex^b^	173 (74.2)	1.27 (0.81–1.99)	0.30		
Alcohol use^b^	157 (67.4)	1.08 (0.69–1.69)	0.74		
CD4 count^b^					
<200	32 (13.7)	Reference			
200–350	60 (25.8)	0.74 (0.35–1.58)	0.44		
>350	141 (60.5)	0.69 (0.35–1.37)	0.29		
WHO stage^b,c^					
I asymptomatic/lymphadenopathy	91 (39.2)	Reference			
II mild local manifestations	87 (37.5)	1.12 (0.62–2.01)	0.71		
III systemic manifestations	52 (22.4)	1.09 (0.57–2.08)	0.80		
IV AIDS-defining illness	2 (0.9)	0.78 (0.10–5.91)	0.81		
Nutritional status^b^					
Adequate nutrition	185 (79.4)	Reference			
Mild to moderate malnutrition	46 (19.7)	1.27 (0.18–9.19)	0.81		
Severe malnutrition	2 (0.9)	1.31 (0.70–2.43)	0.40		
Too sick to work, past 3 months^b,c^	41 (17.6)	0.38 (0.09–1.57)	0.18		
Taking TB treatment, past 3 months^b,c^	12 (5.2)	0.56 (0.08–4.05)	0.57		
Disclosure of HIV status^c,d^	101 (43.7)	1.12 (0.71–1.75)	0.63		
ART use^b^					
None	196 (84.1)	Reference		Reference	
Research clinic	1 (0.4)	0.45 (0.20–0.99)	0.05	0.42 (0.19–0.94)	0.04
Outside clinic	36 (15.5)	0.66 (0.29–1.54)	0.34	0.68 (0.29–1.59)	0.37

*aHR* adjusted hazard ratio, *HR* hazard ratio

^a^Likelihood ratio χ^2^ = 17.70, degrees of freedom = 6, *p* = 0.007

^b^Time-dependent covariate. Note that although only one participant started ART at the research clinic immediately upon enrollment, 25.5 % of included follow-up visits were contributed by participants taking ART at the research clinic and 9.4 % by participants taking ART at an outside clinic

^c^Data were missing for WHO stage (2 visits), being too sick to work (225 visits), TB treatment (221 visits), and disclosure (21 visits). The total of included follow-up visits was 2,667

^d^Disclosure was defined as informing anyone other than the counselor or medical staff (e.g., family, partner, friend, other) about one’s HIV infection

### ART Initiation

Sixty-eight participants, including 30 MSM, 8 heterosexual men, and 30 women started ART at the research clinic. These participants did not differ with respect to nutritional status, CD4 count, WHO stage, co-trimoxazole use, TB treatment, prior ART use, or disclosure of status at baseline. Two MSM were lost to follow-up immediately after starting ART, and eight participants (four women, three MSM, and one heterosexual male) initiated ART the month before the study closed and had no on-treatment follow-up.

The 58 participants included for analysis of ART outcomes were followed for 140.6 person-years, with a median of 856 days per person on treatment (interquartile range [IQR], 583–1,276 days). There was no difference in follow-up time on ART by patient group (KW χ^2^ = 0.30, *p* = 0.86). After ART initiation, one MSM (4.0 %) died of sepsis at 10 months and one woman (3.8 %) died of tuberculosis at 3 months. The mortality rate was 1.4 (95 % CI, 0.4–5.7) per 100 person-years, with no difference between groups (χ^2^ = 0.29, *p* = 0.87 by log-rank test). Three MSM, two heterosexual men, and two women had been on tuberculosis treatment at ART initiation, and three MSM were diagnosed with active tuberculosis 130, 377, and 632 days after ART initiation. Four MSM (16.0 %) and four women (15.4 %) were lost to follow-up, and eight MSM (32.0 %) and one heterosexual man (14.3 %) transferred care. The overall rate of loss to follow-up was 5.7 (95 % CI, 2.8–11.4) per 100 person-years, with no difference between groups (χ^2^ = 1.27, *p* = 0.53 by log-rank test).

Median adherence did not differ across groups, at 98.8 % (IQR, 90.1–99.8 %) for MSM, 99.5 % (IQR, 91.9–100 %) for heterosexual men, and 99.2 % (IQR, 96.4–100 %) for women (KW χ^2^ = 3.17, *p* = 0.20). There was no significant difference in ever having a late refill (72.0 % for MSM, 57.1 % for heterosexual men, 50.0 % for women; FE *p* = 0.29). However, 40 % of MSM had adherence <95 %, versus 28.6 % of heterosexual men and 11.5 % of women (FE *p* = 0.047).

### ART Outcomes

Outcomes after 6–12 months of ART were assessed in 47 (81 %) of 58 participants included in the above analysis. The 11 participants in whom outcomes could not be assessed either died (*n* = 2), were lost to follow-up (*n* = 4), or transferred care (*n* = 2) before the outcome window or had taken ART for <6 months (*n* = 3). On-treatment weight was assessed a median of 310 days after ART initiation (range, 219–364 days) and on-treatment CD4 count was assessed a median of 314 days after ART initiation (range, 219–364 days). Assessment timing did not differ by group (KW χ^2^ = 0.37, *p* = 0.83 for weight gain; KW χ^2^ = 0.56, *p* = 0.75 for CD4 increase).

At assessment, fewer men than women had gained weight (36.4 % of MSM and 50.0 % of heterosexual men vs. 73.7 % of women, FE *p* = 0.06) or had a CD4 count increase (72.7 % of MSM and 16.7 % of heterosexual men vs. 79.0 % of women, FE *p* = 0.01). Table 3 presents linear regression analyses of weight gain per year and CD4 increase per year as clinical outcomes after ART initiation. Mean weight gain was −0.2 kg/year for MSM (standard deviation [SD], 3.4 kg/year) and 4.1 kg/year for heterosexual men (SD, 7.5 kg/year) vs. 3.1 kg/year for women (SD, 4.9 kg/year). The mean CD4 count increase was 56.9 cells/year for MSM (SD, 130.2 cells/year) and −4.5 cells/year for heterosexual men (SD, 56.5) vs. 158.0 cells/year for women (SD, 196.9 cells/year). Lower weight gain among MSM relative to women remained significant after adjustment for potential confounders, including tuberculosis. Although CD4 count increases were lower in MSM and heterosexual men relative to women in unadjusted analysis, this difference was not significant in adjusted modeling.

**Table 3:** Weight gain and CD4 count increase per year on ART, *n* = 47

Characteristic	*N* (%)	Weight gain in kg/year				CD4 count increase in cells/μL			
		Beta (95 % CI)	T statistic (*p* value)	Adjusted Beta^a^ (95 % CI)	T statistic (*p* value)	Beta (95 % CI)	T statistic (*p* value)	Adjusted Beta^b^ (95 % CI)	T statistic (*p* value)
Risk group									
Female	19 (40.4)	Reference		Reference		Reference		Reference	
Heterosexual males	6 (12.8)	1.0 (−3.4 to 5.4)	0.46 (0.65)	−0.6 (−5.6 to 4.3)	−0.26 (0.80)	−162.5 (−309.7 to −15.3)	−2.23 (0.03)	−149.5 (−336.2 to 37.2)	−1.62 (0.11)
MSM	22 (46.8)	−3.3 (−6.3 to −0.3)	−2.25 (0.03)	−3.5 (−6.9 to −0.2)	−2.12 (0.04)	−101.1 (−199.6 to −2.7)	−2.07 (0.04)	−90.0 (−219.1 to 39.1)	−1.41 (0.17)
Age group^c^									
18–24 years	5 (10.6)	Reference		Reference		Reference		Reference	
25–34 years	25 (53.2)	−3.3 (−8.2 to 1.5)	−1.38 (0.17)	−4.7 (−8.9 to −0.5)	−2.24 (0.03)	3.8 (−160.8 to 168.4)	0.05 (0.96)	−31.7 (−187.3 to 123.8)	−0.41 (0.68)
35 and older	17 (36.2)	−2.2 (−7.3 to 2.8)	−0.89 (0.38)	−5.8 (−10.3 to −1.4)	−2.66 (0.01)	−31.4 (−202.3 to 139.5)	−0.37 (0.71)	−55.6 (−224.5 to 113.3)	−0.67 (0.51)
Marital status									
Married	7 (14.9)	Reference		Reference		Reference		Reference	
Single	30 (63.8)	−5.9 (−9.6 to −2.1)	−3.16 (0.003)	−4.7 (−8.4 to −0.9)	−2.52 (0.02)	−9.3 (−141.0 to 122.3)	−0.14 (0.89)	−49.1 (−186.6 to 88.3)	−0.72 (0.47)
Widowed, separated, or divorced	10 (21.3)	−1.9 (−6.3 to 2.5)	−0.88 (0.38)	−3.6 (−8.6 to 1.5)	−1.43 (0.16)	139.6 (−15.0 to 294.1)	1.82 (0.08)	33.8 (−158.8 to 226.5)	0.36 (0.72)
Has dependents	23 (48.9)	3.1 (0.4 to 5.9)	2.27 (0.03)	1.1 (−2.0 to 4.2)	0.74 (0.46)				
Prior treatment^c^	17 (36.2)	0 (−3.0 to 3.1)	0.00 (1.0)	−1.2 (−4.0 to 1.7)	−0.82 (0.42)	−84.1 (−182.2 to 14.0)	−1.73 (0.09)	−96.6 (−205.5 to 12.3)	−1.80 (0.08)
Second-line regimen^c^	2 (4.3)	9.7 (3.0 to 16.3)	2.94 (0.005)	7.6 (0.9 to 14.4)	2.29 (0.03)	−101.5 (−340.8 to 137.7)	−0.85 (0.40)	−46.5 (−306.3 to 213.4)	−0.36 (0.72)

N.B. Educational achievement, monthly earnings, transactional sex, alcohol use, disclosure of HIV status, baseline CD4 count category, baseline WHO stage, baseline nutritional status, baseline illness, and baseline TB treatment were not associated with either weight gain or CD4 count gain at *p* < 0.10 in bivariable analysis and so are not presented

*Beta* difference relative to the reference category

^a^R^2^ = 0.48, adjusted R^2^ = 0.35

^b^R^2^ = 0.28, adjusted R^2^ = 0.13

^c^Collected at the pre-ART baseline

## Discussion

In this small pilot study of HIV care provision to most-at-risk populations in coastal Kenya, both heterosexual men and MSM were less likely than women to report having disclosed their HIV status, had ART counseling, or ever taken ART at enrollment, indicating a low level of engagement with health services in the community. MSM were retained at similar rates to those of heterosexual male and female participants, with ≥80 % retention at 12 months in all three groups. Participants receiving ART through the research clinic were more likely to be retained in care than those not receiving ART, and younger participants were more likely to be lost to follow-up, regardless of patient group. Of note, these barriers experienced by our clinic population (i.e., pre-ART status, younger age) also pertain to other populations of HIV-1-infected adults [22–24].

While men and women had similar health status at ART initiation, MSM had the lowest overall adherence and poor weight gain during treatment. Although three MSM developed tuberculosis after ART initiation, weight gain was not associated with tuberculosis. Both MSM and heterosexual men had suboptimal CD4 counts compared to women, but these differences were not significant in adjusted analysis. These findings support our general impression that among adults reporting high-risk sexual practices, MSM may face greater barriers to ART adherence that could reduce treatment response. Because our population of heterosexual men was very small, it is difficult to draw conclusions from their data.

The role of gender in antiretroviral adherence and response to therapy has varied in different settings and populations. In developed countries, a systematic review found little difference in treatment outcome by gender, although women were thought to have worse adherence and more frequent treatment interruptions than men, possibly due to higher rates of adverse reactions [25]. In contrast, several studies conducted in Africa have reported that men have worse clinical outcomes, lower adherence rates, and lower retention in care than women [26–32]. In this respect, our findings are in accordance with an emerging body of evidence. Several reasons for sub-optimal outcomes among African men have been proposed, including biologic factors such as differences in drug metabolism, toxicities, and opportunistic infection rates, as well as later presentation to care [28–30, 32]. However, these factors have not fully explained poor clinical outcomes among men. One study noted that men who presented for care with their spouses had better outcomes, suggesting that married men may face fewer barriers to care [32]. Additional study of sociobehavioral factors has been proposed, including a review of sociocultural and policy differences between programs in resource-limited settings and resource-rich settings [28–30].

A key difference in sociocultural milieu and policy between many resource-limited and resource-rich settings is service delivery to MSM. Adult same-sex behavior is criminalized and socially unacceptable in many African countries, including Kenya [2]. Stigma, discrimination, and social isolation may seriously undermine the ability of criminalized groups such as MSM to engage in and adhere to care [6, 7]. Evidence suggests that MSM suffer from poor access to HIV-1 testing and prevention services, fear of health-care seeking, denial of care, and even blackmail [5, 6]. These problems may be exacerbated among MSM who rely on sex work for their living, given the increased stigma this entails. It is noteworthy that we identified poor ART outcomes despite a research program specifically oriented towards engaging MSM in care, with counselors trained in male sexual health. Because the vast majority of ART programs in Africa do not specifically identify MSM among their male patients nor provide services targeted to these men’s needs, it is unknown to what degree poor outcomes among African male patients in general may be related to MSM identity.

With the recent success of the HPTN 052 trial, in which ART reduced HIV-1 transmission within discordant couples by an estimated 96 % [13], there is growing interest in prevention in positives and “find, test, link, and retain in care” models [33–35]. The success of such approaches will depend critically on whether HIV-1-infected persons can engage in care, adhere to therapy, and remain in care [8]. It is unclear why the MSM in our study had worse overall adherence and poor weight gain on treatment than female cohort participants. Both groups were similar with respect to age, education, and income levels, and both frequently reported transactional sex work and alcohol use. While several clinic attendees have complained of poor access to adequate food and housing, we did not collect data on food intake or ability to prepare food. Qualitative research regarding African men’s experiences using ART has identified concerns about lack of control and the ability of health care staff to maintain confidentiality [36], but has not addressed other factors that may influence clinical outcomes. Key research priorities recently identified for MSM in Africa include increasing adherence to biomedical interventions and promoting structural interventions to improve access to care [37].

To our knowledge, this is the first study to specifically report on ART outcomes among African MSM, who have only recently become a focus for HIV prevention and treatment programs. However, this study has several important limitations. First, the sample size and follow-up time were limited in this small pilot program. In particular, there were very few heterosexual men in our cohort, so comparisons with this group lack statistical power. Second, the clinic focused on recruitment of men and women reporting high-risk sexual behavior, including anal sex and transactional sex work. As such, these results may not be generalizable to other settings. Third, stigma due to the criminalization of adult same-sex behavior may have led to bias in the reporting of sexual risk behavior and the misclassification of MSM as heterosexual men. Fourth, our ability to track patients after missed visits was restricted to patients taking ART due to limited resources, and we were unable to determine outcomes for participants who were lost to follow-up; in general, we note that mortality was uncommon within the retained cohort, loss to follow-up was not associated with CD4 count or WHO stage, and HIV care was available at several non-research facilities in the clinic catchment area. Fifth, weight and CD4 count were not all assessed at the same time-point, and plasma viral load was unavailable. Despite these limitations, we feel it is important to share our experience providing care to this highly stigmatized group.

## Conclusion

We have found that in a research cohort targeting most at-risk populations in Kenya, participating men, including MSM, were less likely than women to have disclosed their HIV status, had ART counseling, or ever taken ART in the community prior to enrollment. Although men and women had similar health status at ART initiation, MSM had lower adherence and a less robust on-treatment weight gain when compared to women attending the same clinic. Although this study could not definitively address differences in clinical outcomes between MSM and other patients, our results suggest that further research into healthcare disparities affecting African MSM is warranted. Effective interventions to address barriers to access, adherence, and retention among African men are needed, with specific attention to monitoring outcomes among MSM.
